# Evaluation of clinical and laboratory characteristics and factors affecting mortality in 500 hospitalized COVID-19 patients: A retrospective study

**DOI:** 10.15537/smj.2022.43.11.20220641

**Published:** 2022-11

**Authors:** Petek Ş. Konya, Neşe Demirtürk, Derya Korkmaz, Havva Tünay, Elif Betül Koşar

**Affiliations:** *From the from the Department of Infectious Disease and Clinical Microbiology, Afyonkarahisar Health Sciences University, Afyonkarahisar, Turkey.*

**Keywords:** COVID-19, mortality, favipiravir

## Abstract

**Objectives:**

To evaluate the clinical and laboratory characteristics of COVID-19 patients admitted to Afyonkarahisar Health Sciences University, Afyonkarahisar, Turkey, and to determine the factors affecting mortality.

**Methods:**

A total of 500 patients who were diagnosed with COVID-19 between 19th of March and 30th of September 2020 in Afyonkarahisar Health Sciences University, Faculty of Medicine, Pandemic Service, Afyonkarahisar, Turkey, were retrospectively investigated for this study. These individuals’ prognoses, demographic, clinical, laboratory, and radiological information were examined and recorded retrospectively. Comparisons were carried out between the characteristics of patients with a prognosis of death and those who recovered.

**Results:**

Of the 500 definite COVID-19 cases included in the study, 53.8% were male and the mean age was 57.6±15.1 (18-88 years). The most common comorbidities were hypertension and diabetes mellitus. A total of 45 (9%) patients developed mortality. Factors such as advanced age, male gender, shortness of breath, fever at admission, comorbid conditions such as hypertension, diabetes mellitus, cardiovascular diseases, lymphopenia, high C-reactive protein, high D-dimer, and high ferritin in the laboratory were found to be important risk factors for mortality. Treatments such as hydroxychloroquine, favipiravir, and lopinavir/ritonavir were not found to have lower mortality rates than one another.

**Conclusion:**

Considering these elements when assessing patients and adjusting the course of treatment according to the recommendations of the most recent guidelines may lower mortality.

**A** wide clinical range, from asymptomatic outpatients to critically unwell patients needing intensive care unit follow-up, is seen in COVID-19 disease caused by SARS-CoV-2. Risk factors for a severe course have been identified since the pandemic’s start. As a result, it is especially important to monitor individuals at risk of developing a serious illness and dying. To lower the mortality rate, it is critical to identify high-risk individuals as soon as possible.^1^

As a result, the purpose of this study was to establish the factors affecting mortality and to retrospectively assess and diagnose the clinical and laboratory characteristics of COVID-19 patients admitted to our hospital.

## Methods

A total of 500 individuals tested positive for SARS-CoV-2 and were subsequently given a conclusive COVID-19 diagnosis at the Pandemic Service of the Faculty of Medicine, Afyonkarahisar, Turkey. Between March 19 and September 30, 2020, were retrospectively investigated for this study. The definitive diagnosis was made by using RT-PCR to identify the SARS-CoV-2 virus in oropharyngeal and nasopharyngeal swabs. Analyses were carried out on the complete blood count, liver and kidney tests, lactate dehydrogenase (LDH), D-dimer, ferritin, and C-reactive protein (CRP). A thoracic computed tomography (CT) was carried out in all patients except for pregnant patients. These individuals’ prognoses, demographic, clinical, laboratory, and radiological information were examined and recorded retrospectively. Comparisons were carried out between the traits of patients whose prognosis were death and those whose prognosis was the cure. Patient’s files contained demographic information on the patients, while the hospital database had information on laboratory results and radiological images.

Radiological findings included bilateral ground glass opacities, predominantly peripherally located multilobar and subsegmental consolidations, linear opacities, “cobblestone” appearance, and “inverted halo sign.” Other findings such as pleural fluid, cavitation, and lymphadenopathy were defined as atypical involvement.^2^ In our study, radiological findings were classified as typical and atypical.

The guidelines of the Ministry of Health’s COVID-19 Adult Patient Therapy Guide were followed when determining the need for hospital follow-up and the antiviral treatment options and dosages. Transferred to the critical care unit were patients with a respiratory rate >30/minute, progression of dyspnea, and SpO2<90% in room air despite therapy.^3^

The study excluded patients admitted to the intensive care unit, pediatric patients, and patients who were COVID-19 probable cases but whose SARS-CoV-2 PCR test came out negative.

The Ethics Committee of Afyonkarahisar Health Sciences University, Faculty of Medicine, Pandemic Service, Afyonkarahisar, Turkey and the Ministry of Health of Turkey approved this study (date: August 21, 2020, No.: 2020/360).

### Statistical analysis

The Statistical Package for the Social Sciences, version 22.0 (IBM Corp., Armonk, NY, USA) package program was carried out for statistical analysis. Descriptive statistical methods (mean, standard deviation [SD], median, frequency, percentage, minimum, and maximum) were carried out to evaluate the study data. The Shapiro-Wilk test evaluated the conformity of quantitative data to normal distribution. Pearson Chi-square test and Fisher-Freeman-Halton test were caried out to compare qualitative data. A *p*-value of <0.05 was considered significant.

## Results

In this study, 500 patients with a positive SARS-CoV-2 PCR test and a definitive diagnosis of COVID-19 between March 19 and September 30, 2020, were examined. A total of 45 (9%) patients developed mortality. It was determined that 455 patients were discharged with healing, 23 patients resulted in mortality after transfer to intensive care, and 22 were mortal during their follow-up in the ward.

Of the 500 definite COVID-19 cases included in the study, 231 (46.2%) were female, 269 (53.8%) were male, and the mean age was 57.6±15.1 (18-88 years). When the distribution of 45 mortal patients according to gender was analyzed, it was found that there were 38 male and 7 female patients, and mortality was significantly higher in the male gender (*p*≤0.001). The mean age of the patients who resulted in mortality was 72.2 (57-94 years). In patients who did not develop mortality, the mean age was 61.3 years, and age was considered an important risk factor for mortality.

Cough was the most common presenting symptom in 293 (58.6%) patients. A part from this, myalgia was observed in 166 (33.2%) patients, dyspnea in 148 (29.5%) patients, fever in 120 (24%) patients, and sore throat in 76 (15.2%) patients, and headache in 81 (16.1%) patients. Patients with dyspnea (*p*≤0.001) and fever (*p*=0.05) on admission had a significantly higher mortality rate than other patients. The distribution of the patient’s symptoms is shown in Table 1.

**Table 1:** - Symptoms of the patients on admission.

Symptoms	Total	Mortality (n=45)	Non-mortality (n=455)	*P*-values
Cough	293 (58.6)	35 (77.0)	258 (56.7)	0.16
Myalgia	166 (33.2)	10 (22.2)	156 (34.2)	0.55
Shortness of breath	148 (29.5)	32 (71.1)	116 (25.4)	0.001
Fever	120 (24.0)	20 (44.4)	100 (21.9)	0.05
Headache	81 (16.1)	4 (8.8)	77 (16.9)	0.19
Sore throat	76 (15.2)	2 (4.4)	74 (16.2)	0.64
Nausea and vomiting	71 (14.1)	7 (15.5)	64 (14.0)	0.67
Diarrhea	50 (10.0)	5 (11.1)	45 (9.8)	0.19

Values are presented as a number and percentage (%).

The most common comorbidities were hypertension (35.2%), followed by diabetes mellitus (22%), chronic obstructive pulmonary disease (COPD; 13.4%), cardiovascular diseases (5.8%), and renal failure (2.6%). A total of 13 (2.8%) of the patients were pregnant at presentation. When the mortality and non-mortality groups were compared in terms of comorbidities, significant differences were found for hypertension (62.2%-32%), diabetes mellitus (42.2%-20%), cardiovascular diseases (22.0%-4.1%) (*p*≤0.001, *p*=0.05), but not for COPD (*p*=0.67) and renal failure (*p*=0.13).

When the laboratory parameters of the patients were analyzed, leucopenia was found in 68 (13.6%) patients and leucocytosis in 45 (9%) patients. Lymphopenia was found in 103 (20.5%) patients, while lymphocyte count was normal in 397 (79.5%) patients. Thrombocytopenia was found in 120 (24%) patients, and D-dimer level was above 1000 µg/l in 81 (16.2%) patients. Lactate dehydrogenase levels were high in 253 (50.6%) patients, and CRP level was above 100 mg/L in 132 (26.5%) patients. When ferritin values were analyzed, 99 (19.7%) patients had values above 500 mg/L. When the neutrophil/lymphocyte ratio was analyzed, it was found to be above 3.2 in 300 (60%). The mortality rate of patients with lymphopenia (*p*≤0.001), elevated CRP (*p*=0.05), elevated D-dimer (*p*=0.05), and ferritin (*p*=0.006) on admission were significantly higher than the other patients. While leukocyte count, neutrophil/lymphocyte ratio, platelet count, and LDH level did not differ significantly between the mortal and non-mortal groups. The laboratory parameters of the patients are shown in Table 2.

**Table 2:** - Laboratory parameters of the patients.

Findings	Total	Mortality (n=45)	Non-mortality (n=455)	*P*-values
Lymphopenia (<800)/	103 (20.5)	27 (60.0)	76 (16.7)	0.001
Ferritin >500mcg/L	99 (19.7)	21 (46.6)	78 (17.1)	0.05
D-dimer >1000 ng/ml	81 (16.2)	17 (37.7)	64 (14.0)	0.05
CRP >100mg/L	132 (26.5)	32 (71.1)	100 (21.0)	0.006
Thrombocytopenia (<150.000/mm3)	120 (24.0)	15 (33.3)	105 (23.0)	0.55
PNL/L >3.2	300 (60.0)	35 (77.8)	265 (58.2)	0.67
LDH > 250 IU/ml	253 (50.6)	33 (73.3)	220 (48.3)	0.19
Leukocytosis (>10.000)	45 (9.0)	15 (33.3)	30 (6.5)	0.16
Leucopenia	85 (17.0)	5 (11.1)	80 (17.5)	0.13

Values are presented as a number and percentage (%). CRP: C-reactive protein, PNL/L: polymorphous leukocytes/lymphocyte, LDH: lactate dehydrogenase

Thorax CT was carried out in 454 (90.8%) patients, and thorax CT was found normal in 55 (11%) patients. Typical involvement in COVID was found in 360 (72%) patients, while atypical involvement was found in 41 (8.2%) patients. Of the patients with a mortal course, 7 (15.5%) had atypical involvement on thorax CT, and 38 (84.4%) had typical involvement. The mortality rate of patients with typical involvement was significantly higher than those with atypical involvement (*p*≤0.001).

Antiviral treatments were given to the patients following the guidelines of the Ministry of Health, Turkey. When the antiviral drugs used were analyzed, it was determined that 132 (26.4%) patients treated with hydroxychloroquine (HCQ), 314 (62.8%) patients received favipiravir, and 41 (8.2%) patients treated with HCQ + favipiravir, and 13 (2.6%) patients treated with lopinavir/ritonavir. When the antiviral treatments given to the patients with the mortal course were analyzed, 9% of the patients who treated with HCQ, 7.3% of the patients who treated with HCQ + favipiravir, and 9.5% of the patients who treated with favipiravir resulted in mortality, and statistically, no significant difference was found between the antiviral treatments received by the patients with the mortal outcome (*p*=0.16). Mortality rates according to treatment options are shown in Table 3.

**Table 3:** - Treatment options in patients with mortality.

Variables	Hydroxychloroquine	Hydroxychloroquine + favipiravir	Favipiravir	Lopinavir/ritonavir	*P*-value
Mortality	12 (9.0)	3 (7.3)	30 (9.5)	0 (0)	0.16
Non-mortality	120 (91.0)	38 (92.7)	284 (90.5)	13 (100)	

Please note that values are presented as n (%).

## Discussion

In our study, factors such as advanced age, male gender, shortness of breath, and fever at admission, comorbid conditions such as hypertension, diabetes mellitus, cardiovascular diseases, lymphopenia, high CRP, high D-dimer, and high ferritin in the laboratory were found to be important risk factors for mortality.

In our investigation, it was discovered that older people and men had greater death rates. The mortality rate was around 5 times higher in men than in women, and patients who died on average were 72 years old. In a research published in the literature, it was discovered that age, gender, and men’s characteristics were risk factors for mortality.^3^ According to certain research, women are less prone to viral infections than males and have stronger macrophage, neutrophil activation, and superior antibody production.^4^

The most common symptoms of COVID-19 are fever, dry cough, and shortness of breath. Some patients may also experience myalgia, nasal congestion, sore throat, headache, arthralgia, and diarrhea.^5^ In a study by Fu et al^6^ involving 3600 patients, fever was the most common symptom in 83% of patients, cough in 60%, fatigue in 38%, and the mortality rate was significantly higher in patients with fever, fatigue, and headache. On the other hand, fever and cough were the 2 most common symptoms with a rate of 82.1% for fever and 45.8% for cough, and a significant association with mortality was found in Tian et al’s^7^ study in 262 patients. In our study, the cough was the most common symptom with a rate of 46.3%, followed by fever in 29.5%, sore throat in 27.5%, malaise in 26.8%, myalgia in 21.5%, arthralgia in 18.8%, headache in 16.8% and dyspnea in 10.7%. The lower rate of symptoms such as fever and cough compared to other studies was associated with observing mild to moderate cases. When the symptoms were compared in the mortal and non-mortal groups, a significant difference was found in fever (*p*=0.001) and dyspnea (*p*=0.05), whereas no significant difference was found in other symptoms.

The most common comorbidity in our study was hypertension (35.2%), followed by diabetes mellitus (22%), COPD (13.4%), and cardiovascular diseases (5.8%). Comorbidity in COVID-19 patients is a factor that significantly increases mortality.^8^ While 70% of the patients included in our study had comorbidity, all of our patients with mortality had one or more comorbidities. When the mortality and non-mortality groups were compared in terms of comorbidities, a significant difference was found for hypertension (62.2% - 32%), diabetes mellitus (42.2% - 20%), and cardiovascular diseases (22% - 4.1%, *p*≤0.001). In contrast, no significant difference was found between COPD and renal failure. A meta-analysis of 34 studies on comorbidities affecting mortality, diabetes mellitus, COPD, cardiovascular disease, and hypertension were important risk factors for comorbidity. In contrast, no significant relationship was found between acute cardiac failure, renal failure, and mortality.^9^ The results of the studies in the literature were similar to our study.

Laboratory parameters have an important place in the diagnosis and prognosis of COVID-19. Thrombocytopenia and lymphopenia are the most common findings in complete blood count. Elevated D-dimer levels support coagulopathy and are important in the severe disease course. Inflammatory markers are increased during COVID-19, and CRP has an important place in predicting the prognosis of the disease.^10^ In a study carried out on patients with the severe course, Li et al^11^ found that the level of lymphopenia, increased D-dimer, and elevated ferritin were the most important laboratory indicators of prognosis. In our study, when the laboratory parameters of the patients were analyzed, lymphopenia (*p*≤0.001), elevated CRP (*p*=0.05), D-dimer (*p*=0.05), and ferritin (*p*=0.006) were significantly different in the mortal and non-mortal groups.

Thoracic CT plays a complementary role in diagnosing viral pneumonia, and the severity of pneumonia provides valuable information for the prognosis of patients.^12^ A computerized tomography scan was carried out in the majority (90.8%) of the patients we included in the study, and all patients with mortality had pulmonary involvement. While 360 (72%) of the patients had typical involvement in COVID-19, 41 patients (8.2%) had atypical involvement. Of the patients with a mortal course, 7 (15.5%) had atypical, and 38 (84.4%) had typical involvement in thorax CT. The mortality rate of patients with typical involvement was significantly higher than those with atypical involvement (*p*≤0.001). In our study, the microbiological diagnosis was also carried out in patients without typical radiological findings, which are common in COVID-19 patients in the literature. No other reason was found to explain the radiological findings in these patients. Therefore, it should be kept in mind that patients without typical radiological findings may also have COVID-19. Still, it should be taken into account that mortality is less in these patients compared to those with typical involvement.

Hydroxychloroquine is a drug developed in the mid-20^th^ century to treat malaria. It has been used in treating autoimmune diseases due to its immunomodulatory effects on various cytokines, including interleukin-1 (IL-1) and IL-6.^13^ Hydroxychloroquine was shown to be effective against SARS-CoV-2 in invitro studies, but its use was discontinued in later studies due to its lack of antiviral activity against SARS-CoV-2 and its cardiotoxic effects.^14^ In our center, 132 (26.4%) patients were treated with HCQ, and 12 (9%) were mortal. Favipiravir is a purine analog that inhibits the RNA-dependent RNA polymerase of influenza and other RNA viruses and is approved in Japan for treating influenza.^15^ Regarding its potential role in COVID-19, favipiravir has in vitro activity against SARS-CoV-2. However, it is unclear whether sufficient drug levels can be achieved in vivo to inhibit SARS-CoV-2.^16^ In a prospective, multicenter study involving 240 COVID-19-positive adult patients with moderate disease from China, favipiravir was compared with umifenovir in treatment. The clinical cure rate on day 7 was significantly higher in the favipiravir group (71.4%) than in the umifenovir group (55.8%, *p*=0.019).^17^ In our study, 314 (62.8%) patients received favipiravir, and 41 (8.2%) patients received HCQ + favipiravir treatment. In our study, mortality developed in 9.5% of patients receiving favipiravir and 7.3% receiving HCQ + favipiravir.

The US Food and Drug Administration has approved the protease inhibitor lopinavir/ritonavir for the treatment of HIV. It has been demonstrated in vitro that lopinavir/ritonavir inhibits the replication of SARS-CoV-1 and MERS-CoV. According to a study on SARS-CoV-1 patients, those who received lopinavir/ritonavir had lower rates of acute respiratory distress syndrome and mortality.^18^ Additionally, lopinavir/ritonavir and supportive care were evaluated in a research to treat COVID-19, and no appreciable difference was discovered in terms of clinical recovery duration, viral clearance, or mortality.^19^ Since it falls under group B of the Ministry of Health’s treatment recommendations, lopinavir/ritonavir has been included in our country’s treatment options, particularly for pregnant patients. Only pregnant patients in our trial received lopinavir/ritonavir, and no fatality was noted.

None of these antiviral medications are suggested per the current recommendations. When the antiviral therapies that the patients who died got were examined in our study, there was no discernible difference in mortality (*p*=0.16). Numerous meta-analyses have demonstrated that these medications are ineffective, even though their effects could not be assessed because there was no control group in our study that did not receive antiviral medications.^20-22^

Current guidelines recommend nirmatrelvir/ritonavir, monoclonal antibodies in mild outpatients, remdesivir, baricitinib, and tofacitinib in hospitalized moderate to severe cases.^23^ It is important to access current treatments to reduce mortality.^24^

### Study limitation

A standard treatment could not be carried out in patients. It can be explained by adhering to the treatment regimes regulated according to the Ministry of Health guides starting from the first period of COVID-19 disease.

In conclusion, our study found a strong relationship between the risk of death and the presence of concomitant conditions such as hypertension, diabetes mellitus, cardiovascular diseases, lymphopenia, high CRP, raised D-dimer, and ferritin at the time of presentation. Treatments such as HCQ, favipiravir, and lopinavir/ritonavir were not found to have lower mortality rates than one another. Considering these elements when assessing patients and adjusting the course of treatment according to the recommendations of the most recent guidelines may lower mortality.
